# Hyperoside alleviates myocardial ischemia-reperfusion injury in heart transplantation by promoting mitochondrial fusion via activating the Stat3-Tom70-Opa1 pathway

**DOI:** 10.3389/fphar.2025.1566674

**Published:** 2025-09-04

**Authors:** Jincheng Hou, Hongwen Lan, Chenghao Li, Zihao Wang, Qiang Zheng, Kan Wang, Tixiusi Xiong, Yixuan Wang, Jiawei Shi, Nianguo Dong

**Affiliations:** ^1^ Department of Cardiovascular Surgery, Union Hospital, Tongji Medical College, Huazhong University of Science and Technology, Wuhan, China; ^2^ Key Laboratory of Organ Transplantation, Ministry of Education, NHC Key Laboratory of Organ Transplantation, Key Laboratory of Organ Transplantation, Chinese Academy of Medical Sciences, Wuhan, China

**Keywords:** hyperoside, ischemia-reperfusion injury, mitochondrial fusion, oxidative stress, heart transplantation

## Abstract

**Background:**

Myocardial ischemia–reperfusion injury (IRI) is the major cause of primary graft dysfunction in heart transplantation, which is characterized by mitochondrial dysfunction. Hyperoside is a bioactive compound that has been reported to have pharmacological potential for cardiac and mitochondrial protection. Here, we investigated the protective effect of hyperoside during myocardial IRI and identified the underlying mechanisms.

**Methods:**

In this study, we established IRI in an *in vivo* murine heterotopic heart transplantation model and an *in vitro* hypoxia–reoxygenation cell model. Inflammatory responses, oxidative stress level, mitochondrial function, and cardiomyocyte apoptosis were evaluated.

**Results:**

We found that hyperoside pretreatment alleviated through reducing MDA content, LDH activity, TUNEL positive cells, serum cTnI level, Bax protein expression and the level of inflammatory cytokines, and increasing SOD activity and Bcl-2 protein expression. Furthermore, hyperoside pretreatment improved Opa1-mediated mitochondrial fusion, upregulated mitochondrial ATP content and downregulated NADP^+^/NADPH and GSSG/GSH ratios. Opa1 inhibitor blunted the protective effects of hyperoside. Mechanistically, Co-immunoprecipitation experiments showed the binding property between Tom70 and Opa1, siRNA knockdown, AAV-mediated loss-of-function and gain-of-function approaches suggested that hyperoside-promoted Opa1-mediated mitochondrial fusion required the upregulation of Tom70.

**Conclusion:**

Collectively, we demonstrated for the first time that hyperoside administration alleviates myocardial IRI by promoting Opa1-mediated mitochondrial fusion *in vivo* and *in vitro*. The Tom70-Opa1 pathway was essential for cardioprotective effects of hyperoside treatment. The results in our study indicated that hyperoside or promotion of mitochondrial fusion might be a new potential option for the prevention and treatment of IRI in heart transplantation.

## 1 Introduction

Heart transplantation remains the most effective treatment for patients with end-stage heart failure. Despite advances in transplant technologies, ischemia–reperfusion injury (IRI) is still one of the prominent challenges in organ transplantation (Coulson et al., 2005; Capuzzimati et al., 2022; Smith et al., 2019; Zhai et al., 2013; Wang et al., 2019). Severe myocardial IRI, which usually occurs in the hearts subjected to prolonged cold ischemia time, is associated with primary graft dysfunction and mortality following heart transplantation (Chambers et al., 2017; Kwon et al., 2022; Tang et al., 2021; Valero-Masa et al., 2020; John et al., 2019). Therefore, new strategies that alleviate myocardial IRI in donor hearts with prolonged cold ischemia time during heart transplantation are needed.

Hyperoside (Hyp), a flavonoid glycoside extracted from various fruits and herbs, such as *Hypericiaceae*, *Rosaceae*, *Platycodon*, and *Rhododendraceae* (Wang et al., 2022). Hyp is one of the major components of Chinese traditional patent medicines, has several cytoprotective properties included anti-apoptosis, anti-inflammatory, antioxidation, and anticancer property (Chen et al., 2023; Li W. et al., 2014; Choi et al., 2011; Ku et al., 2015; Charachit et al., 2022). Moreover, accumulating evidence demonstrates that Hyp pretreatment can potentially alleviate IRI multiple organs, especially in heart (Wu et al., 2019; Hou et al., 2016; Liu et al., 2012; Shi et al., 2019; Han et al., 2015). Nevertheless, due to the significant differences between cold IRI and warm IRI, the role of Hyp during IRI in heart transplantation needs further investigation.

Mitochondrial dynamics refers to the dynamic balance between mitochondrial fusion and fission. It is well established that during the process of IRI, mitochondrial division increases and fusion decreases, leading to a mitochondrial fission–fusion imbalance (Maneechote et al., 2022; Vongsfak et al., 2021). Dynamin-related protein 1 (Drp1) downregulation or optic atrophy 1 (Opa1) upregulation has been reported to exert cardioprotective effects during cardiac IRI (Du et al., 2022; Zhuang et al., 2021). Additionally, previous research has indicated that Hyp can inhibit adriamycin-induced mitochondrial dysfunction through suppressing mitochondrial fission both *in vivo* and *in vitro* (Chen et al., 2017). However, it remains unknown whether mitochondrial dynamics plays a crucial role in the Hyp-mediated protection of donor heart. Given the established role of STAT3 in regulating mitochondrial gene expression and fusion-related signaling pathways, we hypothesized that hyperoside may exert its protective effect via the activation of STAT3 and subsequent transcriptional regulation of mitochondrial regulators such as Tom70 and OPA1.

The present study investigated the effects of Hyp during IRI in heart transplantation and explored the mechanism underlying the role of mitochondrial fusion in mediating the cardioprotective effects of Hyp.

## 2 Materials and methods

### 2.1 Reagents

**FIGURE 1:**
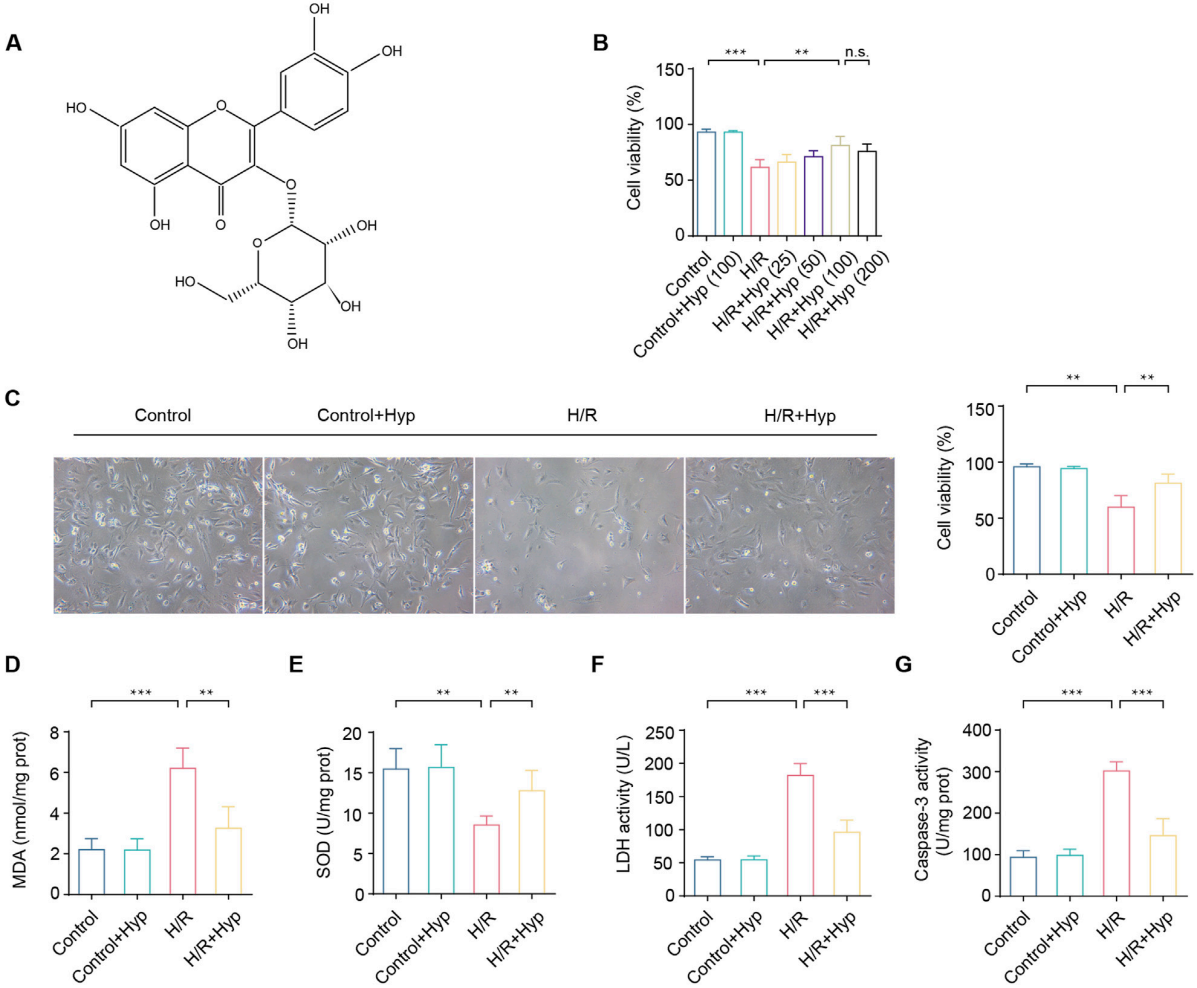
Hyp pretreatment attenuates H/R injury in NRCMs. **(A)** Chemical structure of Hyp. **(B)** Cell viability of NRCMs by Hyp pretreatment at 25, 50, 100, 200 μmol/L for 12 h. **(C)** Cell viability (100 μmol/L), **(D)** MDA content, **(E)** SOD activity, **(F)** LDH activity, and **(G)** Caspase-3 activity were detected in NRCMs subjected to H/R injury. Data are shown as the mean ± SD. *P < 0.05, **P < 0.01, ***P < 0.001, and n.s. no significant difference for indicated comparisons.

Hyp (C21H20O12, purity >98%, Figure 1A) and MYLS22 (purity >98%) were purchased from MCE (Shanghai, China). Commercially available kits to evaluate malondialdehyde (MDA) content, superoxide dismutase (SOD) activity, glutathione peroxidase (GPX) activity, ATP content, ROS production, and bicinchoninic acid (BCA) protein assay kit were purchased from Beyotime Biotechnology (Shanghai, China). Interleukin-6 (IL-6), monocyte chemoattractant protein-1 (MCP-1), Tumor necrosis factor (TNF-α), lactate dehydrogenase (LDH), and cardiac troponin I (cTnI) were determined by ELISA kits (Nanjing Jiancheng Bioengineering Institute, China). ELISA kits (Sigma, United States) were used to assay NADP^+^/NADPH ratio, GSSG/GSH ratio, citrate synthase (CS) activity and isocitrate dehydrogenase (IDH) activity. Radioimmunoprecipitation assay (RIPA) lysis buffer (50 mM Tris-HCl, 150 mM sodium chloride, 1% Triton X-100, 1% sodium deoxycholate, and 0.1% sodium dodecyl sulphate) and protease inhibitor were procured from Servicebio (Wuhan, China). Antibodies against Bax (32,503), GAPDH (ab8245), Tom70 (ab289977), phosphorylated-Stat3 (p-Stat3, ab30647), Stat3 (ab119352), Opa1 (ab42364), Drp1 (ab184247), mitofusin 1 (Mfn1, ab221661), and mitofusin 2 (Mfn2, ab124773) were obtained from Abcam (Cambridge, UK). Antibodies against Bcl-2 (26593-1-Ap) was purchased from Proteintech (Wuhan, China), while those against β-tubulin (2146) was obtained from Cell Signaling Technology (Danvers, MA, United States).

### 2.2 Neonatal rat cardiomyocytes isolation and culture

Neonatal rat cardiomyocytes (NRCMs) were isolated from Sprague–Dawley rats 1–3 days after birth. Ventricle tissue was rapidly harvested, put in ice-cold Hanks solution to remove blood, minced, and incubated with digestion solution including 0.1% trypsin and 1 mg/mL collagenase type II at 4 °C overnight. The next day, the digested tissue was transferred to a dish and collected into a conical tube. The digestion solution was added to the cardiac tissue and incubated at 37 °C for 15 min with gentle shaking. The supernatant containing the cells was transferred to fresh Dulbecco’s modified Eagle’s medium (DMEM) containing 20% fetal bovine serum (FBS), and the undigested cardiac tissue was resuspended in the digestion solution. The digestion step was repeated five times, and the cell-containing supernatant was collected. After purification using Percoll density-gradient centrifugation, NRCMs were cultured in a humidified incubator with 5% CO2 at 37 °C. The culture medium contained 10% FBS and 1% penicillin/streptomycin.

### 2.3 *In vitro* hypoxia–reoxygenation model

A hypoxia–reoxygenation (H/R) model was established in line with a previous report (Zhu et al., 2019). The vehicle or various concentrations of Hyp were added for 12 h before H/R induction. In order to induce H/R, the cell culture medium was replaced in the UW solution, and the cells were removed to a hypoxia incubator bag at 4 °C, 0.1% O2. After 24 h of hypoxia, the cells were returned to normal culture condition (95% air, 5% CO2), and the medium was changed to complete DMEM (containing 10% FBS and 1% penicillin/streptomycin) for another 6 h to mimic the reperfusion phase *in vitro*. Cells cultured in the above normal cell culture conditions were used as normoxia control.

### 2.4 Animals experimental design

All animal experimental procedures were approved by the Ethic Committee on Animal Care of Union Hospital, Tongji Medical College, Huazhong University of Science and Technology (Approval number:3086). Male C57BL/6 mice, 8–12 weeks old, weighing 22–28 g, were purchased from Huafukang Experimental Animal Center, China. All animals were housed under pathogen-free conditions on a 12-h light/dark cycle at 23 °C ± 1 °C, and were fed with a regular pellet diet *ad libitum* during this period.

The mice were received with the vehicle or Hyp via intragastric gavage (i.g.) once daily for 1 week before surgery. In this study, Hyp was dissolved in the vehicle (0.5% sodium carboxymethyl cellulose) and pretreated with 25, 50 or 75 mg/kg/day based on previous studies (Wu et al., 2019; Shi et al., 2019). A single dose of 1 × 1011 vector genomes of adeno-associated virus (AAV) serotype 9 were transfected via tail vein injection into each mouse for the study of Tom70 overexpression or knockdown. The genes carried by the AAV included Tom70 (AAV-Tom70), Tom70-interfering sequence (AAV-sh-Tom70), GFP control gene (AAV-GFP) or control-interfering sequence (AAV-sh-Con). Mice were started to use for further experiments after 4 weeks of AAV injection. All the AAV used in the study were constructed and purchased from Dianjun Biotechnology (Shanghai, China).

### 2.5 Donor heart retrieval and preservation

The retrieval of donor heart was performed as described previously (Loganathan et al., 2015). Briefly, after preparation of the perfusate, the mice were anesthetized by inhalation of isoflurane (5% to induce anesthesia, 2.5% to maintain anesthesia). Heparin (400 IU/kg) was injected from inferior vena cava to achieve systemic heparinization. The University of Wisconsin (UW) solution was used to induce cardiac arrest; then, a standard method was used to harvest the heart and it was placed in a UW solution at 4 °C. Donor hearts from mice in the IRI group were subjected to heart transplantation after 24 h cold preservation, while donor hearts from mice in the Control group were transplanted instantly without cold preservation.

### 2.6 Heterotopic heart transplantation

Syngeneic murine heterotopic heart transplantation was performed as described previously (Zheng et al., 2021). Briefly, mice were anesthetized by inhalation of isoflurane (5% to induce anesthesia, 2.5% to maintain anesthesia). Following the opening of the abdomen, the abdominal aorta and inferior vena cava were visible. Then, the recipient’s abdominal aorta was anastomosed to the donor’s ascending aorta, and the recipient’s inferior vena cava was anastomosed to the donor’s pulmonary artery. All processes of heart transplantation were conducted within 1 h.

### 2.7 RNA interference *in vitro*

Control scramble siRNA (Ruibo Biotechnology, Guangzhou, China) or Tom70 siRNA (Ruibo Biotechnology, Guangzhou, China) was transfected into NRCMs for RNA interference. The Tom70 siRNA-targeting sequences used was GTG CTA TAC CGA GGC TAT T. The LipofectamineTM 3000 Transfection Reagent (Thermo Fisher Scientific, United States) was used to start transfection in Opti-MEM for 12 h, then complete DMEM was replaced for another 12 h to prepare for further experiments.

### 2.8 Terminal deoxynucleotidyl transferase dUTP nick end labelling assay

Myocardial apoptosis was evaluated in the left ventricle with Roche terminal deoxynucleotidyl transferase dUTP nick end labeling (TUNEL) assay according to manufacturer’s instructions.

### 2.9 Mitochondrial oxidative stress evaluation and LDH detection

Mitochondrial oxidative stress indicators (i.e., MDA content, SOD and GPX activities) and LDH level were measured using commercially available kits following the manufacturer’s instructions. The serum sample was collected after heart transplantation to analyze the concentrations of cTnI and inflammatory cytokines included IL-6, MCP-1, and TNF-α using commercially available kits. The ATP content, NADP^+^/NADPH ratio, GSSG/GSH ratio, CS activity and IDH activity were evaluated according to manufacturer’s instructions.

### 2.10 Detection of intracellular ROS

We mounted frozen cardiac tissue sections on glass slides using dihydroethidium (DHE) to detect cardiac ROS production during IRI. Microscopy was used to determine the percentage of DHE-stained areas on cryosections stained with DHE for 30 min at 37 °C. Intracellular ROS levels in NRCMs were detected using a Reactive Oxygen Species Assay Kit (ROS Assay Kit, Beyotime, Cat#S0033S) according to the manufacturer’s instructions. After various treatments, cells were incubated with 10 μM DCFH-DA at 37 °C in the dark for 20 min, followed by three washes with serum-free medium. Fluorescence images were captured using a confocal laser scanning microscope, and the intensity of green fluorescence (DCF signal) was used to reflect intracellular ROS levels.

### 2.11 Real-time quantitative PCR

Total RNA was extracted from NRCMs using Trizol reagent (Thermo Fisher Scientific, United States) and subjected to reverse transcription using the RT Reagent Kit (Vazyme Biotech, Nanjing, China) to obtain cDNA. SYBR Green (Vazyme Biotech, Nanjing, China) was used in real-time PCR assays to quantify Tom70 mRNA levels on Applied Biosystems (Thermo Fisher Scientific, United States). The mRNA expression was normalized to GAPDH using the 2−△△ct method. The primers used in this study were Tom70-F 5′-GTG​TTT​AGA​AGA​TGT​CAC​TGC​TG-3′ and Tom70-R 5′-TGG​CTG​AGA​AAT​TAT​GTC​ATC-3′, GAPDH-F 5′-GCA​TCT​TCT​TGT​GCA​GTG​CC-3′ and GAPDH-R 5′- GAT​GGT​GAT​GGG​TTT​CCC​GT-3′. All the primers were designed by AuGCT DNA-SYN Biotechnology Co., Ltd (Wuhan, China).

### 2.12 Western blot analysis

Western blot analysis was performed on samples preserved in liquid nitrogen from the left ventricle. Briefly, the cardiac tissue was crushed and lysed in RIPA lysis buffer mixed with a protease inhibitor cocktail. BCA protein assay kit was used to detect protein concentration. Proteins were separated through sodium dodecyl sulphate-polyacrylamide gel electrophoresis, and transferred onto polyvinylidene difluoride membranes (Millipore, Billerica, MA, United States), blots then incubated with primary antibodies against Bax (1:1000), Bcl-2 (1:1000), Tom70 (1:1000), p-Stat3 (1:1000), Stat3 (1:1000), Opa1 (1:1000), Drp1 (1:1000), Mfn1 (1:1000), Mfn2 (1:1000), β-tubulin (1:25,000), and GAPDH (1:25,000). Following overnight incubation at 4 °C, the membranes were washed in tris buffered saline tween (10 mM Tris-base, 100 mM sodium chloride, and 0.1% tween-20, pH 7.50) and incubated with secondary antibodies for 1 h at room temperature. The protein bands were quantified using the Image Lab software system (Bio-Rad, Hercules, CA, United States).

### 2.13 Co-immunoprecipitation assay

Co-immunoprecipitation (Co-IP) assay was performed as described previously (Jiang et al., 2022). Cells were lysed in IP lysis buffer (Beyotime Biotechnology, Shanghai, China). Anti-Tom70, anti-Opa1, and IgG antibody (Beyotime Biotechnology, Shanghai, China) were incubated with cell lysate overnight at 4 °C. Protein A/G agarose beads (Beyotime Biotechnology, Shanghai, China) were used to pull down antibody-protein conjugates by 4 h of incubation. Then, the beads were washed, boiled, and centrifuged. Finally, the samples were separated through western blotting.

### 2.14 Surface plasmon resonance (SPR) assay

To evaluate the direct binding affinity between Hyp and STAT3 protein, a SPR assay was performed using a Biacore 8K system (GE Healthcare). Recombinant human STAT3 protein (92–95 kDa, 50 μg) was immobilized on a CM5 sensor chip (Cytiva, BR-1005-30) via amine coupling chemistry using an EDC/NHS-based activation protocol. Immobilization was carried out at a flow rate of 10 μL/min using acetate buffer (pH 5.0), followed by blocking with ethanolamine. The final coupling response was approximately 11,202 RU. The interaction assay was conducted using PBS-P+ running buffer (pH 7.4) containing 5% DMSO, and Hyp was diluted into multiple concentrations using serial dilutions in 96-well plates. The analyte was injected at a flow rate of 30 μL/min for 150 s in ascending concentrations, followed by dissociation and regeneration of the chip surface with 10 mM glycine-HCl (pH 2.0) for 5 min. A solvent correction curve was generated using varying DMSO concentrations (4.5%–5.8%) to ensure accurate response unit (RU) calibration. Data were analyzed using Biacore Insight software, and binding kinetics were fitted using a 1:1 Langmuir interaction model.

### 2.15 Cellular thermal shift assay (CETSA)

To investigate the interaction between Hyp and STAT3, a CETSA was performed in NRCMs. NRCMs were pretreated with 100 μM Hyp or an equal volume of DMSO as control for 2 h. After treatment, cells were harvested and resuspended in PBS supplemented with protease inhibitors. Cell suspensions were aliquoted and subjected to thermal challenge using a Veriti 96-Well Thermal Cycler (Thermo Fisher Scientific) for 10 min at 37, 40, 43, 46, 49, 52, 55, 58, and 61 °C, respectively, followed by cooling at room temperature for 10 min. Following the thermal treatment, samples were lysed by three freeze–thaw cycles using liquid nitrogen, and the soluble protein fractions were collected by centrifugation at 12,000 rpm for 15 min at 4 °C. The remaining soluble STAT3 protein was detected by Western blotting, and the band intensities were quantified to assess thermal stabilization of STAT3 by Hyp.

### 2.16 Statistical analysis

Data are expressed as mean ± standard deviation of the mean (SD) values. Differences in the overall mean values between two groups were assessed by two-tailed parametric t-test if the values were normally distributed. A non-parametric Mann–Whitney U test was used to compare non-normally distributed variables between two groups. A significant difference was defined as P < 0.05. Analyses were performed using SPSS 26.0 (IBM Corporation, Armonk, NY, United States).

## 3 Results

### 3.1 Hyp pretreatment attenuates H/R injury in NRCMs

To investigate the cardioprotective effects of Hyp, we first assessed the role of Hyp during H/R injury in NRCMs. As shown in Figure 1B, with the increase of Hyp concentration, the survival rate of NRCMs increased gradually. However, we did not find better therapeutic effects in H/R + Hyp (200) group compared to H/R + Hyp (100) group. In order to avoid a possible effect of drug overdose on the study outcome, Hyp (100 μmol/L) was chosen for subsequent studies *in vitro*. We found that the NRCMs subjected to H/R without Hyp pretreatment showed very low cell viability and SOD activity and very high MDA level and LDH activity, respectively (Figures 1C–F). In contrast, the NRCMs subjected to H/R with Hyp pretreatment showed higher cell viability and SOD activity but lower MDA level and LDH activity (Figures 1C–F). Additionally, pretreatment with Hyp significantly reduced Caspase-3 activity during H/R injury (P < 0.001; Figure 1G). These results suggest that Hyp pretreatment attenuates H/R injury in NRCMs.

### 3.2 Hyp pretreatment alleviates myocardial IRI in heart transplantation

**FIGURE 2:**
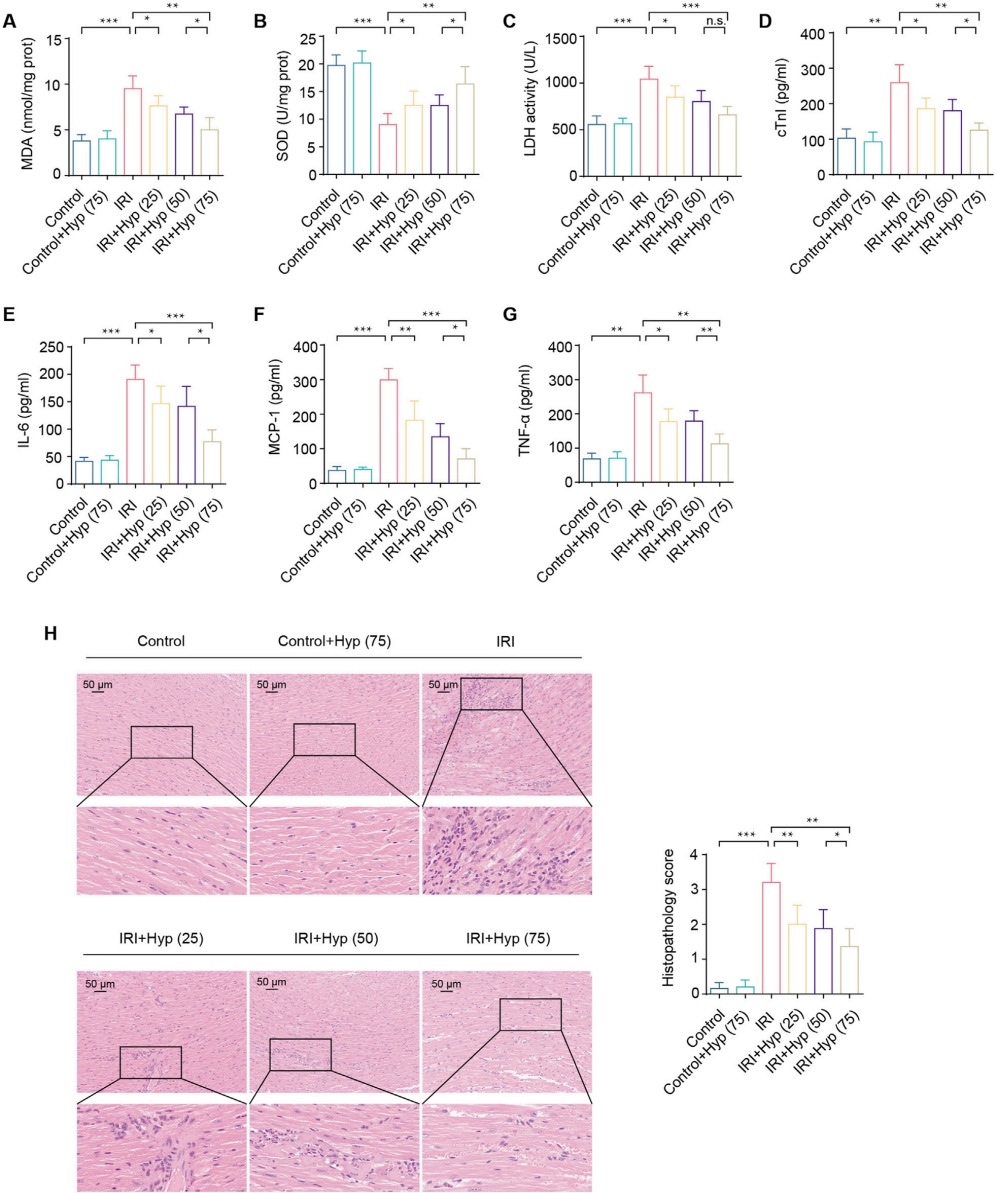
Hyp pretreatment alleviates myocardial IRI in heart transplantation. **(A)** MDA content, **(B)** SOD activity, and **(C)** LDH activity were detected in the hearts subjected to myocardial IRI. The levels of **(D)** cTnI and inflammatory cytokines, including **(E)** IL-6, **(F)** MCP-1, and **(G)** TNF-α were detected in the serum from the heart following myocardial IRI. **(H)** Inflammatory changes in the heart were assessed by H&E staining (magnifcation, × 20; scale bar, 50 μm). Data are shown as the mean ± SD. *P < 0.05, **P < 0.01, and ***P < 0.001 for indicated comparisons.

We next investigated the cardioprotective effects of Hyp in murine heterotopic heart transplantation. There was a marked increase in MDA content and LDH activity and a decrease in SOD activity in the cardiac tissue, indicating that oxidative stress was induced following IRI in heart transplantation (P < 0.001; Figures 2A–C). Hyp administration protected the heart from IRI through reducing in MDA content and LDH activity and increasing in SOD activity, and these effects were most evident in the group of IRI + Hyp (75). Therefore, 75 mg/kg/day Hyp was considered to be the optimal dose for the animal study and was used for subsequent experiments. Myocardial injury was alleviated by Hyp pretreatment as evidenced by the decrease in serum cTnI level (Figure 2D). To further evaluate graft contractile function, transabdominal echocardiography was performed in a subset of mice after heterotopic heart transplantation. Although imaging the grafted heart in the abdominal cavity is technically demanding, measurable ejection fraction (EF) values were obtained. The EF of the IRI + Hyp group (55.6% ± 3.4%) was significantly higher than that of the IRI group (41.3% ± 2.7%), suggesting improved systolic function with Hyp pretreatment.

IRI causes a significant increase in myocardial inflammation. As shown in Figures 2E–G, levels of inflammatory cytokines, including IL-6, MCP-1, and TNF-α, were increased in the serum from the mice following IRI in heart transplantation. Treatment with Hyp protected the grafts from IRI by reducing the release levels of IL-6, MCP-1, and TNF-α. As expected, significant myocardial inflammation was induced by IRI according to H&E staining (Figure 2H). In comparison with the Control group, the Hyp-treated group showed a significant alleviation in myocardial inflammation. Taken together, these results suggest that Hyp pretreatment alleviates myocardial IRI in heart transplantation.

### 3.3 Hyp pretreatment improves mitochondrial function and promotes mitochondrial fusion in heart transplantation

**FIGURE 3:**
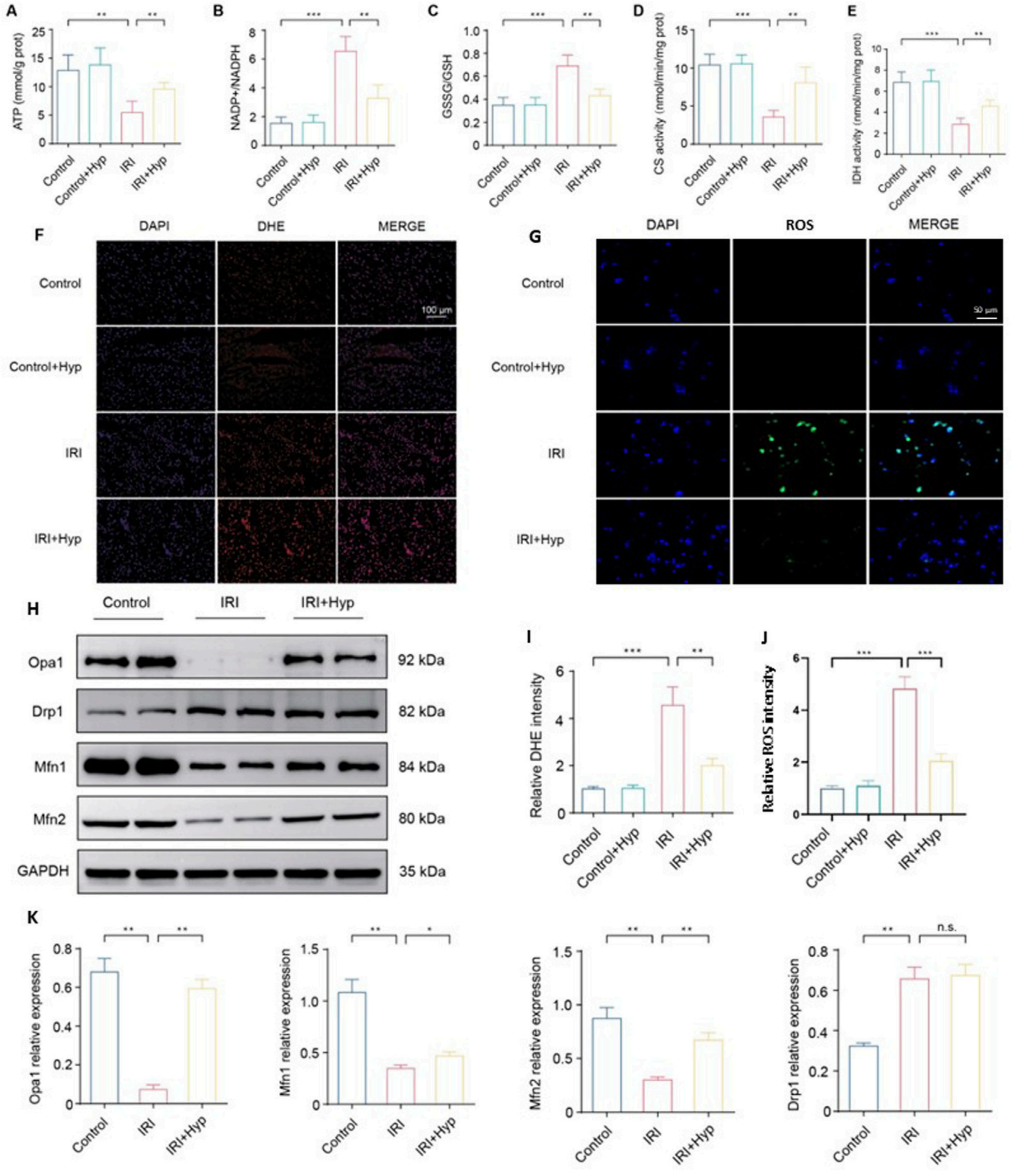
Hyp pretreatment improves mitochondrial function and promotes mitochondrial fusion in heart transplantation. The levels of **(A)** ATP content, **(B)** NADP^+^/NADPH, **(C)** GSSG/GSH, **(D)** citrate synthase activity, and **(E)** isocitrate dehydrogenase activity were detected in the hearts subjected to myocardial IRI by ELISA kits. **(F)** Fluorescence images of ROS production in each group (magnifcation, × 200; scale bar, 100 μm). ROS were subjected to DHE staining (red), and the nuclei of all cardiomyocytes were stained with DAPI (blue). **(G)** Representative confocal microscopy images showing intracellular ROS levels in NRCMs using the DCFH-DA-based ROS Assay Kit. **(H)** Protein expression levels of mitochondrial dynamics–related proteins, including Opa1, Mfn1, Mfn2, and Drp1. **(I)** Quantification of DHE fluorescence intensity. **(J)** Quantification of intracellular ROS fluorescence intensity. **(K)** Quantification of relative expression levels of Opa1, Drp1, Mfn1, and Mfn2. Data are shown as the mean ± SD. *P < 0.05, **P < 0.01, and ***P < 0.001 for indicated comparisons.

Due to the importance of mitochondria in a variety of physiological and pathophysiological processes, we next examined the alterations in mitochondria during myocardial IRI. As shown in Figures 3A–C, myocardial IRI downregulated mitochondrial ATP content and evoked NADP^+^/NADPH and GSSG/GSH ratios. These alterations were attenuated following Hyp pretreatment (P < 0.01). As expected, Hyp pretreatment improved the mitochondrial oxidative phosphorylation during H/R as reflected in the increased activities of CS and IDH (P < 0.01; Figures 3D,E). Moreover, DHE staining revealed elevated ROS levels in cardiac tissue after IRI, which were significantly reduced by Hyp pretreatment (P < 0.01; Figures 3F,I). To further validate the antioxidative effect of hyperoside at the cellular level, we used a DCFH-DA-based ROS assay to detect intracellular ROS levels in NRCMs subjected to H/R injury. As shown in Figure 3G, ROS production was markedly elevated in H/R-treated cells, while hyperoside pretreatment significantly reduced ROS levels, indicating its robust antioxidant efficacy *in vitro* (Figure 3J).

To identify the molecular mechanism of Hyp in mitochondrial function, we investigated the expression of key proteins involved in mitochondrial dynamics. As shown in Figure 3H, Hyp pretreatment increased the protein expression of Opa1, Mfn1, and Mfn2, which regulated the mitochondrial fusion process. However, there was no significant change in Drp1 expression following Hyp pretreatment, indicating that mitochondrial fusion, rather than mitochondrial fission, is likely involved in Hyp cardioprotective effects during IRI. The relative expression levels of these proteins are summarized in Figure 3K. These results suggest that Hyp pretreatment improves mitochondrial function and promotes mitochondrial fusion in heart transplantation.

### 3.4 Hyp alleviates myocardial IRI through Opa1-mediated mitochondrial fusion

**FIGURE 4:**
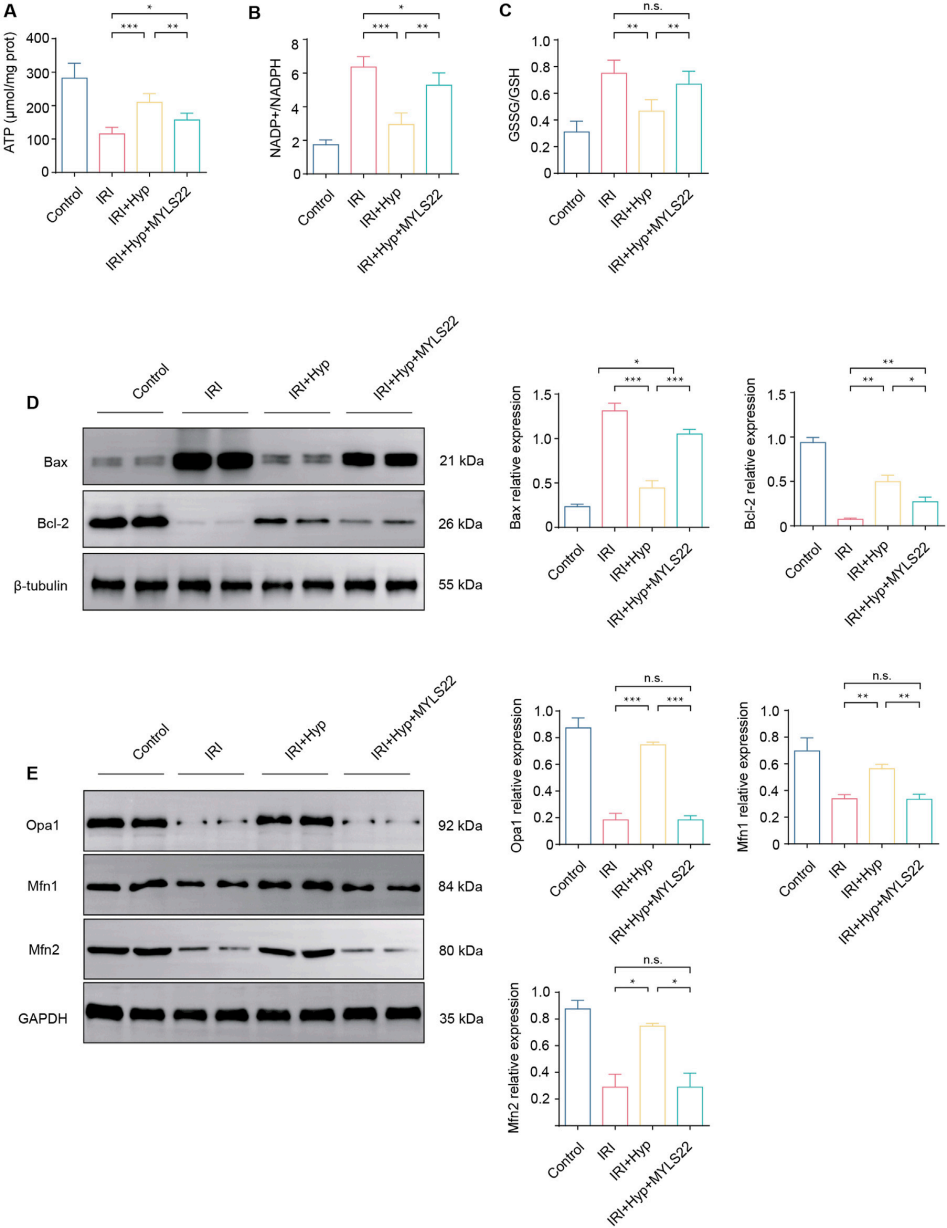
Effects of MYLS22 administration on myocardial IRI after Hyp treatment. The levels of **(A)** ATP content, **(B)** NADP^+^/NADPH, and **(C)** GSSG/GSH were detected in the hearts subjected to myocardial IRI by ELISA kits. **(D)** The expression levels of Bax and Bcl-2 were detected by western blotting. **(E)** The expression levels of mitochondrial fusion–related proteins, including Opa1, Mfn1, and Mfn2. Data are shown as the mean ± SD. *P < 0.05, **P < 0.01, ***P < 0.001, and n.s. no significant difference for indicated comparisons.

Opa1 is an important mitochondrial fusion-associated protein, we next explored whether Opa1-mediated mitochondrial fusion was responsible for cardioprotective effects of Hyp. As shown in Figures 4A–C, administration of MYLS22 (a selective Opa1 inhibitor, 10 mg/kg via intraperitoneal injection 30 min before surgery) abolished the protective effects of Hyp, as reflected in the significant decrease in ATP content, and a significant increase in NADP^+^/NADPH and GSSG/GSH ratios. The anti-apoptotic effects of Hyp were abolished by MYLS22, as demonstrated by the increased expression level of the pro-apoptotic factor Bax, and reduced expression level of the anti-apoptotic factor Bcl-2 (Figure 4D). Moreover, MYLS22 administration decreased the expression of mitochondrial fusion–related proteins, including Opa1, Mfn1, and Mfn2 (P < 0.01; Figure 4E). These results suggest that Opa1-mediated mitochondrial fusion is responsible for cardioprotective effects of Hyp.

### 3.5 Hyp promotes Opa1-mediated mitochondrial fusion via the Stat3-Tom70 signaling pathway

**FIGURE 5:**
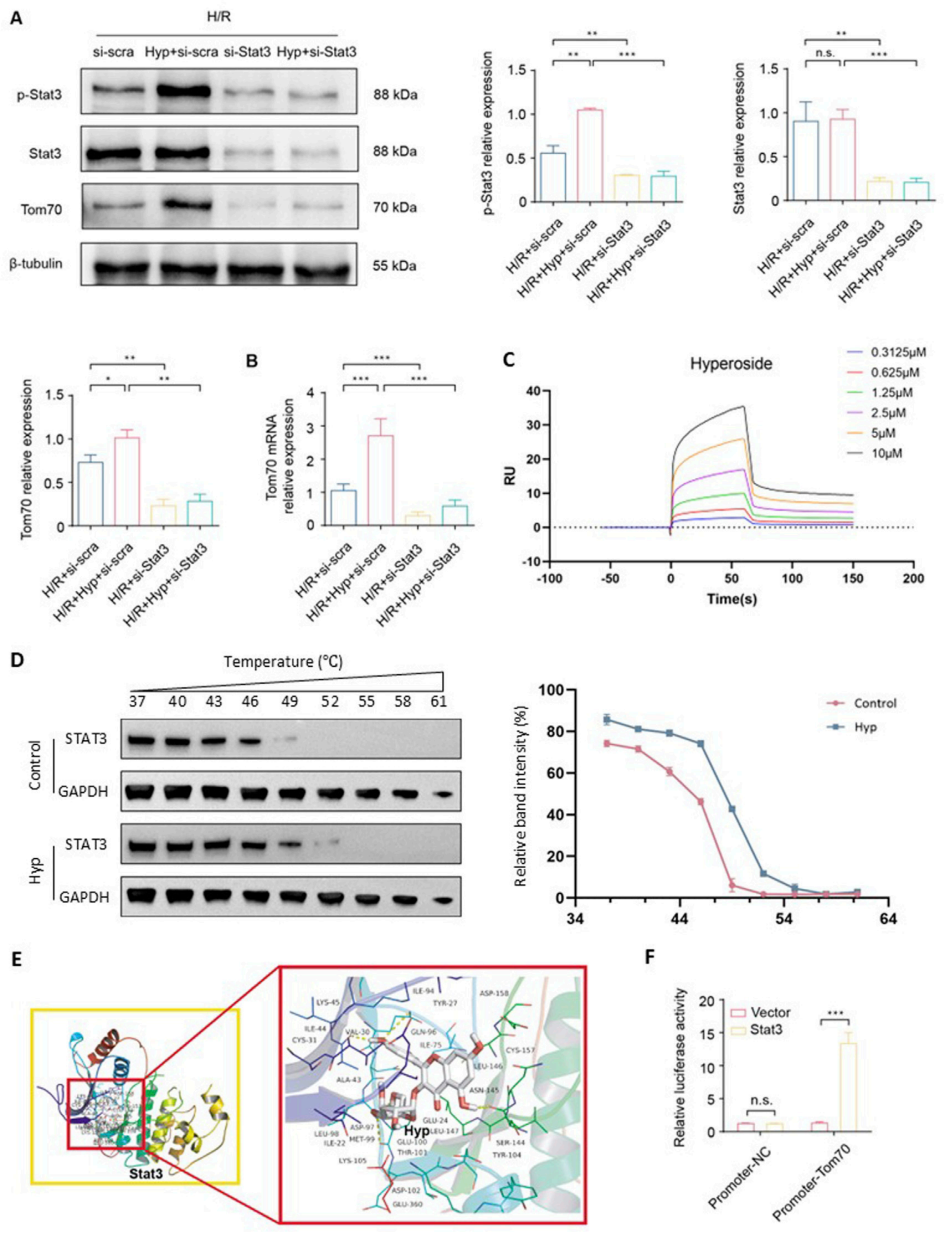
Hyp promotes Opa1-mediated mitochondrial fusion via the Stat3-Tom70 signaling pathway. **(A)** Protein expression levels of Tom70, p-Stat3, Stat3 in NRCMs treated with Hyp and si-Stat3 following H/R. **(B)** Tom70 mRNA expression in NRCMs treated with Hyp and si-Stat3 following H/R. **(C)** SPR analysis showing the direct binding between Hyp and recombinant human STAT3 protein. The sensorgram indicates a dose-dependent interaction, and kinetic analysis yielded K_D_ = 5.72 μM, Ka = 3.81 × 10^4^ (1/Ms), and Kd = 2.18 × 10^−1^ (1/s). **(D)** CETSA showing that Hyp increased the thermal stability of STAT3 protein in NRCMs. Right panel: relative band intensities (%) quantified across a temperature gradient (37 °C–61 °C). **(E)** Binding mode between Hyp and Stat3. **(F)** Dual luciferase assay was conducted to examine the regulatory ability of Stat3 on the Tom70 promoter. *P < 0.05, **P < 0.01, ***P < 0.001, and n.s no significant difference for indicated comparisons.

As a constituent subunit of the translocase of mitochondrial outer membrane (Tom) complex, Tom70 is essential for the mitochondrial transport system and plays important roles in the regulation of mitochondrial function. We found that Tom70 protein expression was markedly increased in the Hyp-treated NRCMs after H/R (P < 0.05; Figure 5A). Meanwhile, we found that the protein levels of p-Stat3 were upregulated (P < 0.01; Figure 5A). Interestingly, Stat3-siRNA significantly downregulates the levels of Tom70 protein, suggesting that Stat3 might regulate the Tom70 protein expression following H/R. The same result was further approved by the quantitative real-time PCR (Figure 5B). To investigate whether Hyp directly interacts with STAT3, we performed SPR analysis, which demonstrated a direct and concentration-dependent binding between Hyp and human STAT3 protein, with a dissociation constant of 5.72 μM (Figure 5C). Furthermore, a CETSA showed that Hyp treatment enhanced the thermal stability of STAT3 in NRCMs (Figure 5D), supporting the physical interaction between Hyp and STAT3 in cells. The Auto Dock Vina binding test indicated that Hyp might favorably bind to Stat3 (maximum binding affinity – 7.5 kcal/mol; Figure 5E). Dual luciferase assay demonstrated that β-catenin could directly bind to the promoter of GS (Figure 5F). In summary, these results suggested that Hyp promotes Opa1-mediated mitochondrial fusion via the Stat3-Tom70 signaling pathway.

### 3.6 The Stat3-Tom70-Opa1 pathway is essential for cardioprotective effects of Hyp

**FIGURE 6:**
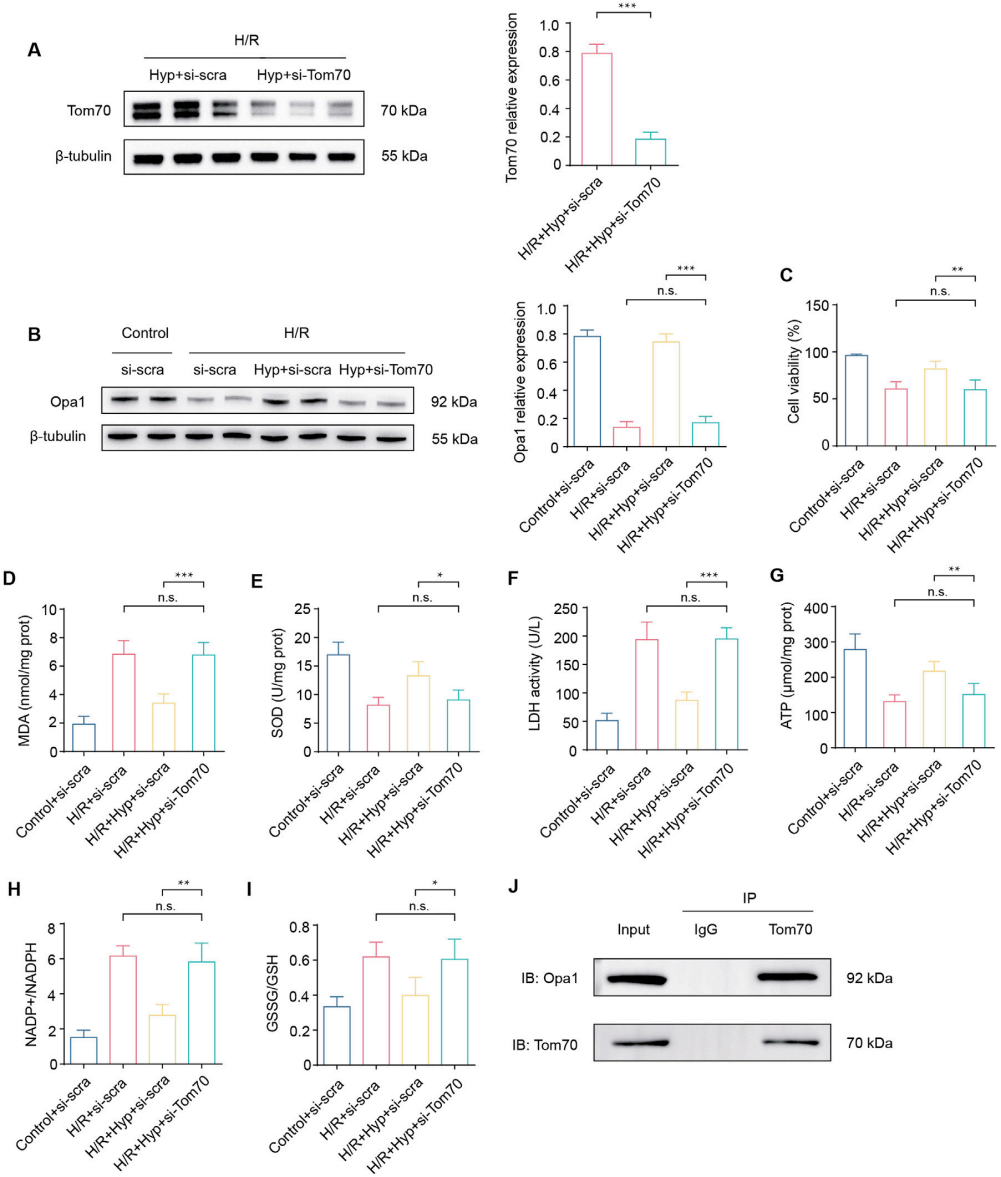
Effects of Tom70 knockdown after Hyp treatment *in vitro*. **(A)** Protein expression levels of Tom70 in NRCMs treated with Hyp following H/R. **(B)** Protein expression levels of Tom70 in NRCMs treated with si-Tom70 following H/R. **(C)** Protein expression levels of Opa1 in NRCMs treated with or without si-Tom70. **(D)** Cell viability, **(E)** MDA content, **(F)** SOD activity, and **(G)** LDH activity were detected in NRCMs. The levels of **(H)** ATP content, **(I)** NADP^+^/NADPH, and **(J)** GSSG/GSH were detected in NRCMs subjected to H/R by ELISA kits. Immunoprecipitation of Tom70 and Opa1. *P < 0.05, **P < 0.01, ***P < 0.001, and n.s no significant difference for indicated comparisons.

In order to investigate the role of Tom70, si-Tom70 was used to knockdown Tom70 in NRCMs (Figure 6A). As shown in Figure 6B, si-Tom70 significantly reduced Opa1 protein expression in NRCMs subjected to H/R. In addition, Tom70 knockdown reduced the cell viability, increased the level of MDA, and decreased the activity of SOD (P < 0.05; Figures 6C–E). The enhancement of LDH activity indicated severe cell damage during H/R under Tom70 knockdown (P < 0.001; Figure 6F). Compared to the IRI + Hyp + si-scra group, Tom70 knockdown downregulated mitochondrial ATP content and increased NADP^+^/NADPH and GSSG/GSH ratios, indicated that Tom70 knockdown abolished the protective effect of Hyp on mitochondrial functions (Figures 6G–I). Previous study has indicated that Tom70 and Opa1 can bind to each other (Li J. et al., 2014). We demonstrated the binding property between Tom70 and Opa1 through Co-IP experiments, and the results suggested that Hyp exert cardioprotective effects through Stat3-Tom70-Opa1 pathway *in vitro* (Figure 6J).

**FIGURE 7:**
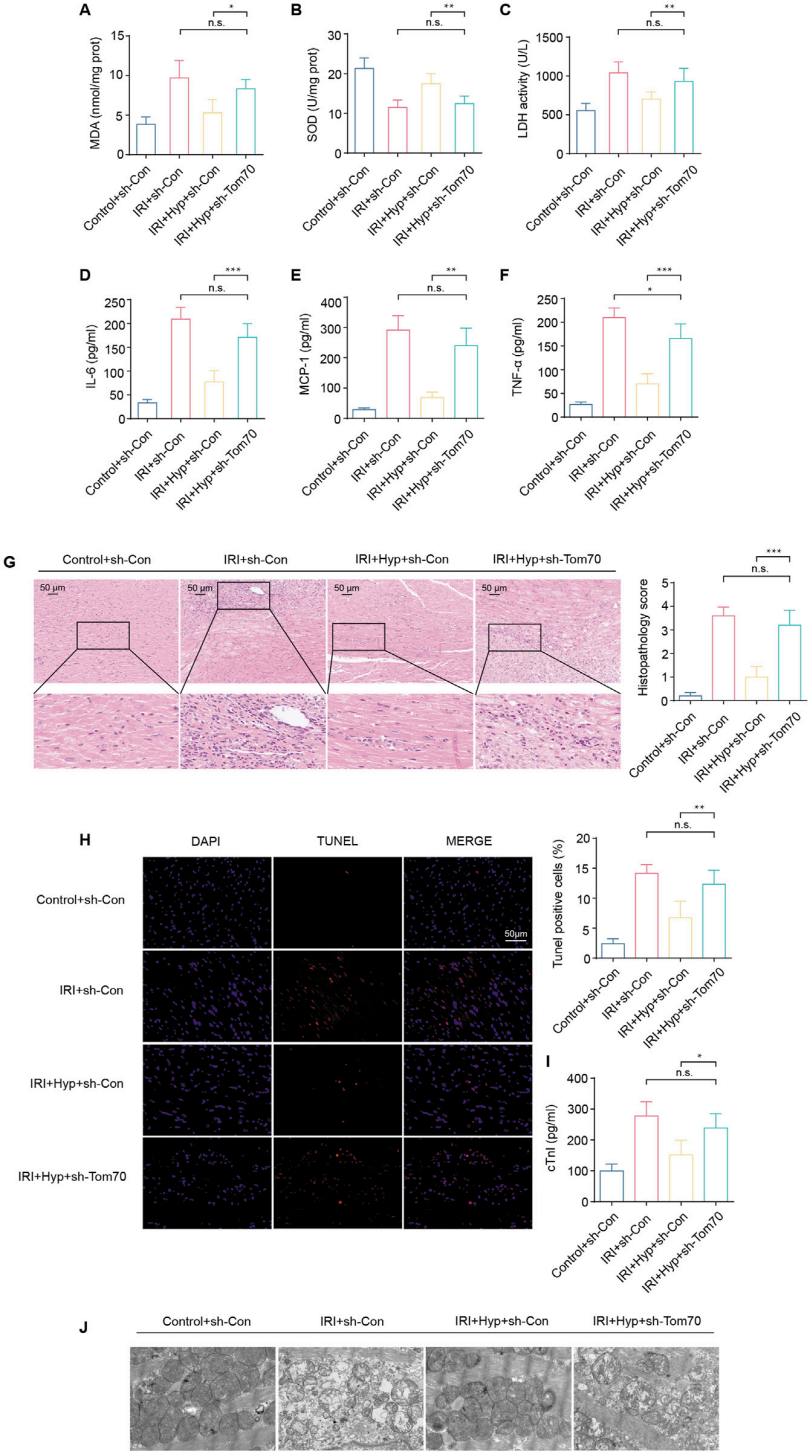
Effects of Tom70 knockdown after Hyp treatment *in vivo*. **(A)** MDA content, **(B)** SOD activity, and **(C)** LDH activity were detected in the hearts subjected to myocardial IRI. The levels of inflammatory cytokines, including **(D)** IL-6, **(E)** MCP-1, and **(F)** TNF-α were detected in the serum from the heart following myocardial IRI. **(G)** Inflammatory changes in the heart were assessed by H&E staining (magnifcation, × 20; scale bar, 50 μm). **(H)** Fluorescence images of TUNEL assay in each group. Apoptotic cardiomyocytes were subjected to TUNEL staining (red), and the nuclei of all cardiomyocytes were stained with DAPI (blue). The apoptotic index was expressed as the percentage of apoptotic cells relative to the total cells. **(I)** Serum cTnI level. **(J)** Representative transmission electron microscopy images. *P < 0.05, **P < 0.01, ***P < 0.001, and n.s no significant difference for indicated comparisons.

**FIGURE 8:**
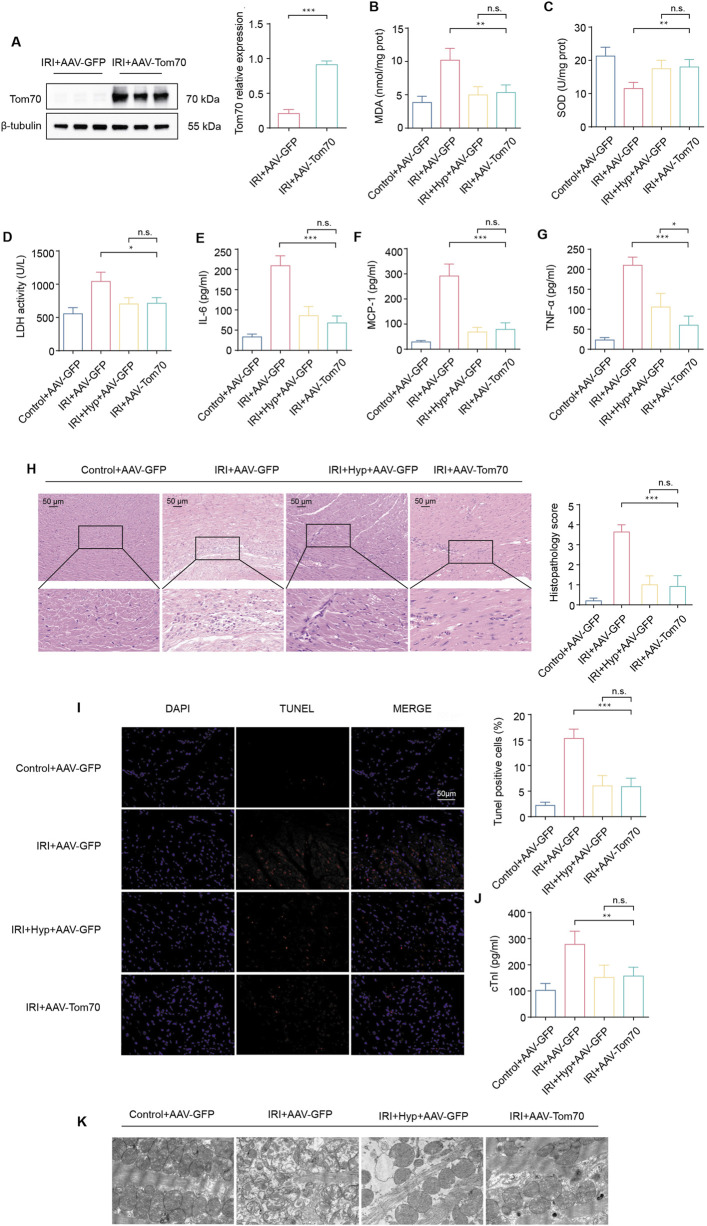
Effects of Tom70 overexpression after Hyp treatment *in vivo*. **(A)** Protein expression levels of Tom70 in hearts treated with AAV-Tom70 following IRI in heart transplantation. **(B)** MDA content, **(C)** SOD activity, and **(D)** LDH activity were detected in the hearts subjected to myocardial IRI. The levels of inflammatory cytokines, including **(E)** IL-6, **(F)** MCP-1, and **(G)** TNF-α were detected in the serum from the heart following myocardial IRI. **(H)** Inflammatory changes in the heart were assessed by H&E staining (magnifcation, × 20; scale bar, 50 μm). **(I)** Fluorescence images of TUNEL assay in each group. Apoptotic cardiomyocytes were subjected to TUNEL staining (red), and the nuclei of all cardiomyocytes were stained with DAPI (blue). The apoptotic index was expressed as the percentage of apoptotic cells relative to the total cells. **(J)** Serum cTnI level. **(K)** Representative transmission electron microscopy images. *P < 0.05, **P < 0.01, ***P < 0.001, and n.s no significant difference for indicated comparisons.

To explore whether Tom70 is essential for the cardioprotective effects of Hyp *in vivo*, we used AAV-mediated loss-of-function approaches. As shown in Figures 7A–F, the administration of sh-Tom70 abolished the protective effects of Hyp, as confirmed by the greater aggravation found in hearts from the IRI + Hyp + sh-Tom70 group compared to those from the IRI + Hyp + sh-Con group according to the MDA content, LDH activity, SOD activity, and the release levels of IL-6, MCP-1, and TNF-α. The reductions in the levels of inflammatory infiltrate, apoptotic cardiomyocytes and serum cTnI following Hyp treatment were markedly blocked by Tom70 knockdown (Figures 7G–I). Moreover, the decrease in mitochondrial damage after Hyp pretreatment was impaired in hearts from the IRI + Hyp + sh-Tom70 group (Figure 7J). We further confirm the role of Tom70 in the cardioprotective effects of Hyp *in vivo*, we used AAV-mediated gain-of-function approaches (Figure 8A). Compared with the IRI + AAV-GFP group, Tom70 overexpression attenuated oxidative stress by decreasing in MDA content and LDH activity and increasing in SOD activity in the IRI + AAV-Tom70 group (Figures 8B–D). Interestingly, no significant differences were found between the IRI + AAV-Tom70 group and IRI + Hyp + AAV-GFP group. Additionally, the results indicated that AAV-Tom70 was not inferior to Hyp in anti-inflammatory effects (Figures 8E–H). Myocardial injury was alleviated by Tom70 overexpression as evidenced by the increase in TUNEL positive cells and serum cTnI level (Figures 8I,J). We further explored the myocardial injury via transmission electron microscope (TEM) at the microstructure level. As shown in Figure 8K, the IRI group with Hyp pretreatment or AAV-Tom70 showed significantly reduced microstructural damage of ischemic cardiomyocyte mitochondria compared to the IRI group without treatment. Taken together, these results suggest that Stat3-Tom70-Opa1 pathway is essential for cardioprotective effects of Hyp.

## 4 Discussion

In the present study, we demonstrated for the first time that Hyp administration inhibits oxidative stress, inflammatory response and myocardial apoptosis by promoting Opa1-mediated mitochondrial fusion *in vivo* and *in vitro*. We found that Hyp pretreatment induced Tom70 expression through, Mechanistically, using *in vitro* and *in vivo* studies, it is verified that Hyp binds to and induces the phosphorylation of Stat3, which then binds to the Tom70 promoter to promote Opa1-mediated mitochondrial fusion. The results in our study indicated that Hyp or promotion of mitochondrial fusion might be a new potential option for the prevention and treatment of IRI in heart transplantation. In the current study, the selected hyperoside doses (25, 50, and 75 mg/kg/day) were based on previously reported effective ranges in rodent models of IRI. While 75 mg/kg/day produced optimal cardioprotective effects, the relatively narrow dose span may limit the exploration of a full dose–response relationship. Future studies involving both lower and higher dose regimens will be necessary to comprehensively define the therapeutic window of hyperoside in the context of myocardial IRI.

A study from Li and colleagues confirmed the cardioprotective effects of Hyp in a Langendorff model, and the results suggested that administration of Hyp significantly improved heart contraction of the ischemic/reperfused isolated rat heart, reduced the infarct size and the level of creatine kinase and LDH. In addition, Hyp treatment remarkably decreased the MDA content and increased the SOD activity as well (Hou et al., 2016). Additionally, Hyp ameliorated doxorubicin-induced oxidative stress in HL-1 cells, increased GSH, SOD and catalase activity, reduced ROS production and MDA content (Chen et al., 2023). Hyp therapy significantly alleviated cell apoptosis through upregulating the Bax protein expression level and downregulating the Bcl-2 protein expression level. Moreover, in a study was related to ischemia/reperfusion-induced acute kidney injury, Hyp pretreatment reduced TUNEL-positive cells and the level of cleaved Caspase-3, attenuated the decreased expression of Opa1, inhibited mitochondrial fission (Wu et al., 2019). In our study, Hyp pretreatment significantly reduced MDA content, LDH activity, TUNEL positive cells, serum cTnI level, and Bax protein expression level, and increased SOD activity and Bcl-2 protein expression level. Furthermore, Hyp pretreatment improved Opa1-mediated mitochondrial fusion. Although Seahorse XF analysis would provide dynamic measurements of mitochondrial respiration parameters such as basal respiration, ATP production, and maximal respiration, this assay was not feasible within the current study due to equipment and time constraints. Instead, we employed a set of validated biochemical assays that serve as widely accepted surrogates for mitochondrial respiratory capacity, including ATP content, citrate synthase and isocitrate dehydrogenase activities, and redox balance markers. These assays, together with mitochondrial ROS measurements, consistently demonstrated that Hyp pretreatment preserves mitochondrial bioenergetics and integrity following H/R or IRI. In future work, we plan to perform Seahorse XF analysis to complement these findings and provide a more comprehensive bioenergetic profile.

Mitochondria are intracellular organelles that are necessary for cellular energy production, metabolism and signaling, cell survival, and apoptosis. Mitochondrial biogenesis and function depend on the rigorous transport system, which imports more than 1,000 types of precursor proteins into the mitochondria (Pfanner et al., 2019; Endo and Yamano, 2009; Neupert and Herrmann, 2007). As the major part of the mitochondrial transport system, Tom complexes consist of different subunits, including Tom20, Tom22, Tom40, and Tom70. A core region in Tom70 protein contributes to the recognition of the internal targeting signals of polytopic membrane proteins (Araiso et al., 2022; Hansson et al., 2008). Moreover, the Tom70-mediated import pathway depends on chaperones, notably Hsp70 and Hsp90 (Fan et al., 2010; Backes et al., 2018). There is recent evidence that Tom70 might play a key role in innate immunity, mitochondrial biogenesis, cellular bioenergetics, and adaptations to stress conditions (Liu et al., 2010; Latorre-Muro et al., 2021; Filadi et al., 2018; Backes et al., 2021; Liu et al., 2022; Kreimendahl and Rassow, 2020). In addition, Tom70 expression is downregulated and aggravates mitochondrial oxidative stress in the process of pathological cardiac hypertrophy (Li J. et al., 2014). Tom70 treatment mitigates mitochondrial fragmentation, reduces reactive oxygen species (ROS) level, and improves cardiac function following myocardial IRI (Pei et al., 2017; Wang et al., 2020). Moreover, Tom70 is involved in the cardioprotective effects of many drugs in previous studies (Wang et al., 2020; Hou et al., 2022). However, the role and mechanism of Tom70 during IRI have not been revealed in the above studies. Xue et al. found that Tom70 knockdown significantly aggravated myocardial IRI and increased mitochondrial calcium overload, while treatment with Tom70 significantly alleviated myocardial IRI, improved mitochondrial function, and relieved mitochondrial calcium overload. Hence, Tom70 exerts these cardioprotective effects by regulating the mitochondrial localization of mitochondrial calcium uptake 1 (MICU1) (Xue et al., 2017). In our study, we found a elevated expression of Tom70 after Hyp pretreatment during myocardial IRI, indicating that Tom70 might play a pivotal role in the process of Hyp therapy. We found that the protective effect of Hyp was diminished by Tom70 knockdown, and myocardial IRI was alleviated by Tom70 overexpression. Our data suggest that STAT3 activation mediates the upregulation of Tom70 at both mRNA and protein levels. While luciferase reporter assays support direct transcriptional regulation, further studies such as chromatin immunoprecipitation (ChIP) are warranted to confirm STAT3 binding to the Tom70 promoter. These findings propose a novel Stat3–Tom70–OPA1 axis that links extracellular stimuli (hyperoside) with mitochondrial fusion and cardioprotection. These results suggest that altered Tom70 expression contributes to myocardial IRI.

Mitochondrial dynamics include mitochondrial fusion and mitochondrial fission, which play an essential role in the maintenance of mitochondrial homeostasis and cell survival. Mitochondrial fusion is a form of mitochondrial morphological change, which occurs in all eukaryotic cells with mitochondria. The process of mitochondrial fusion can finish in as little as 2 min and result in the exchange of mitochondrial DNA, proteins, lipids, and metabolites. In mammalian cells, Mfn1 and Mfn2 are GTPases anchored to the outer mitochondrial membrane and serve as essential effector factors for outer membrane fusion, whereas Opa1 is localized to the inner mitochondrial membrane and mediates inner membrane fusion and cristae remodeling. Importantly, pharmacological activation of mitofusins, particularly Mfn2, has been shown to restore mitochondrial morphology, enhance bioenergetic function, and improve cellular survival in disease models such as Charcot–Marie–Tooth disease type 2A (Franco et al., 2022). These findings underscore the broader therapeutic potential of targeting mitochondrial fusion proteins across diverse pathological conditions. Considering that Hyp treatment in our study upregulated both Mfn1/Mfn2 and Opa1, it is conceivable that strategies aimed at activating mitofusins may complement Opa1-mediated inner membrane fusion to achieve more robust mitochondrial protection in the setting of cardiac ischemia–reperfusion injury. There is substantial evidence of increased mitochondrial fission and reduced mitochondrial fusion in cardiomyocytes during IRI. Large amounts of ROS and intracellular calcium overload following IRI impair mitochondrial function and homeostasis, resulting in mitochondrial fragmentation and cardiomyocyte apoptosis. Therefore, mitochondrial fusion exerts cardioprotective effects by protecting the defective mitochondria by diluting damaged proteins or allowing normal mitochondrial DNA to enter the defective mitochondria. A previous study showed that the expression of mitochondrial fusion proteins was reduced following IRI, while the pharmacological mitochondrial fusion promoter M1 restored the expression of mitochondrial fusion proteins, attenuated mitochondrial dysfunction, preserved cardiac function, and decreased mortality (Maneechote et al., 2019). Moreover, M1 exerts cardioprotective effects by consistently increasing the expression of Opa1, promoting mitochondrial fusion, enhancing mitochondrial respiratory capacity, and reducing mitochondria-derived superoxide production (Ding et al., 2020). In this study, we demonstrated that the expression of mitochondrial fusion–related proteins, including Opa1, Mfn1, and Mfn2, was markedly reduced in the IRI group compared with the Control group, indicating that mitochondrial fusion was reduced in hearts following IRI. Furthermore, myocardial IRI increased the expression of mitochondrial fission-related protein Drp1. As a result of all these processes, excessive mitochondrial fragmentation was generated. In our study, MYLS22, an OPA1 inhibitor, led to reduced expression of not only OPA1 but also MFN1 and MFN2. This may reflect a secondary effect of mitochondrial fusion disruption, where impaired inner membrane fusion induces mitochondrial stress and retrograde signaling that suppress outer membrane fusion proteins. Previous studies have shown that dysfunctional mitochondrial dynamics can trigger mitofusin degradation or transcriptional repression as part of cellular quality control. Further investigations will be necessary to fully delineate the molecular mechanisms connecting OPA1 inhibition with mitofusin downregulation.

The Tom70-Opa1 pathway has been revealed in the process of pathological cardiac hypertrophy (Li J. et al., 2014). Li and colleagues observed a reduction in the expression of Opa1 in Tom70-deficient cells, suggesting that the abnormality in mitochondrial morphology caused by Tom70 reduction was associated with the deficiency in mitochondrial Opa1. The results of Co-IP assays indicated that Tom70 could directly bind to Opa1 and that deficiency in mitochondrial Opa1 mimics the cellular features of pathological cardiac hypertrophy induced by Tom70 deficiency. In our study, we further demonstrated Opa1 as a downstream effector of the Tom70-dependent pathway underlying the cardioprotection of Hyp. The observed reduction in OPA1 expression following Tom70 knockdown may result from impaired mitochondrial protein import, altered mitochondrial proteostasis, or indirect transcriptional mechanisms. As Tom70 functions as a key component of the mitochondrial translocase system, its deficiency may hinder the proper import or stability of OPA1. Our Co-IP data and previous studies in cardiac models support the existence of a functional interaction between Tom70 and OPA1. Further studies are needed to determine whether this regulation occurs at the import, post-translational, or transcriptional level. Compared with the H/R + Hyp + si-scra group, the expression of Opa1 was decreased in the si-Tom70 treatment group, indicating that Opa1 expression was regulated by Tom70. Furthermore, we identified the association between Tom70 and Opa1 using Co-IP assays, and the results suggest that Tom70 can directly bind to Opa1. However, the molecular mechanisms mediating the regulation of Tom70-Opa1 pathway has not yet been entirely elucidated.

Recent advances in alleviating myocardial IRI in donor hearts subjected to prolonged cold ischemia time, including drug treatment, gas therapy, and mitochondrial transplantation, focus on the mitochondria of donor hearts (Zhu et al., 2019; Moskowitzova et al., 2019; Cai et al., 2016; Benke et al., 2021). Similar to previous studies, the results in our study indicated that Hyp or promotion of mitochondrial fusion might be a new potential option for the prevention and treatment of IRI in heart transplantation (Wang et al., 2025). The Stat3-Tom70-Opa1 pathway might account for cardioprotection exerted by Hyp. Although our study employed siRNA-mediated Stat3 knockdown to demonstrate that Stat3 is indispensable for Hyp-induced Tom70 expression and cardioprotection, we acknowledge that this approach does not fully recapitulate the complete and permanent loss of Stat3 achievable by CRISPR-Cas9–mediated knockout. Generating Stat3-null cardiomyocytes via CRISPR-Cas9, or using cardiac-specific Stat3 knockout mice, would provide more definitive genetic evidence. Due to time and resource constraints, these experiments were not performed in the current study, but we plan to incorporate them in future work to further validate our conclusions. However, the specific targets for regulation of Hyp, the precise regulatory mechanism of Stat3-Tom70-Opa1 pathway, require further investigation. Additionally, the cardioprotective effect of Hyp has to be confirmed in a large animal model to translate this novel approach into clinical practice. Although no classical pharmacological positive control was included in this study, we utilized a range of complementary oxidative stress and injury markers to evaluate the cardioprotective efficacy of hyperoside. These include both cellular-level assays and tissue-level readouts, which served as internal functional benchmarks. The consistency across these assays strengthens the validity of our findings. Nevertheless, we acknowledge the importance of including an established therapeutic comparator in both *in vitro* and *in vivo* models to further define the efficacy profile of hyperoside. Future studies will incorporate clinically relevant reference agents to enable more direct translational comparisons.

## 5 Conclusion

**FIGURE 9:**
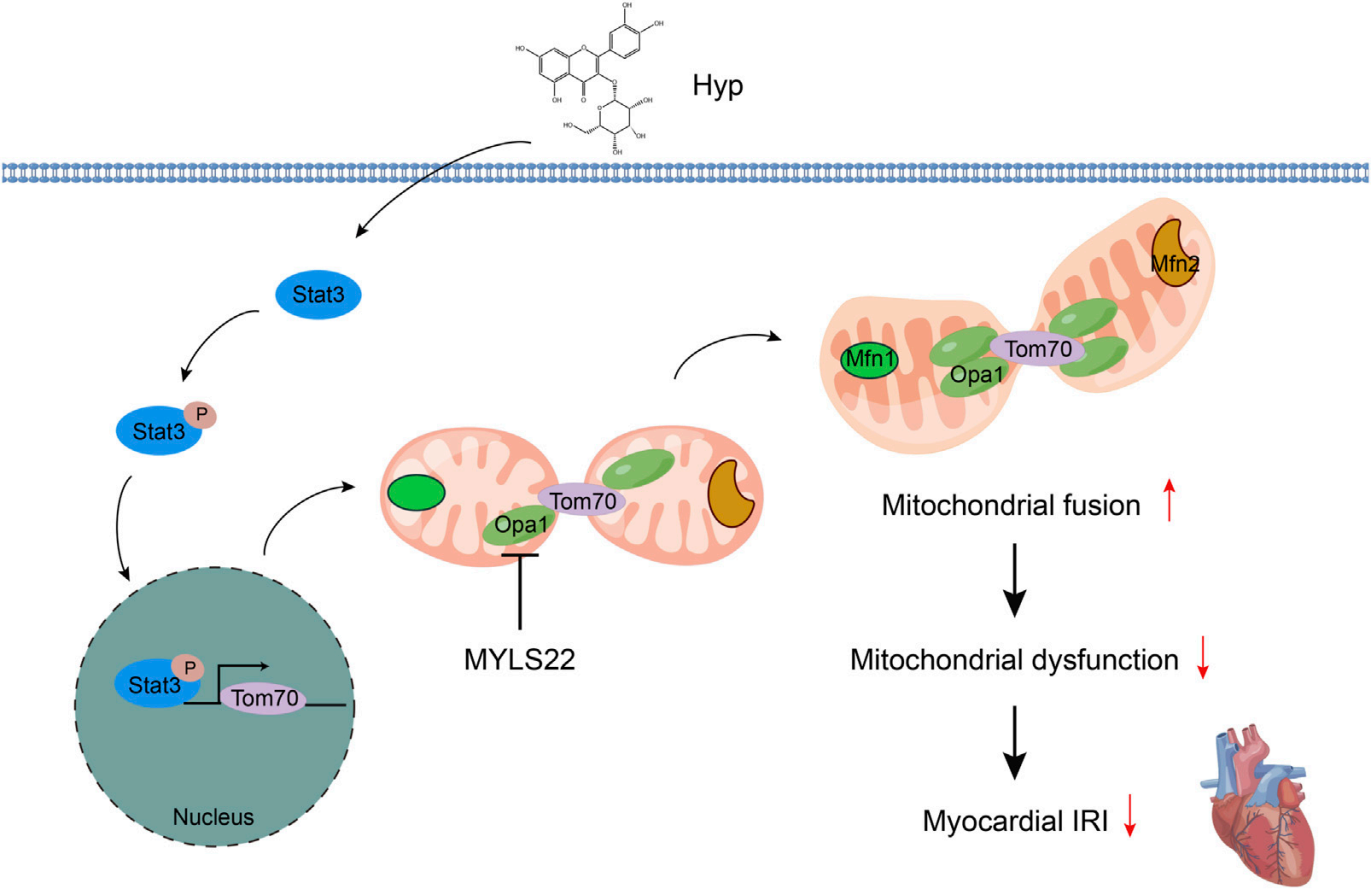
Schematic diagram of Hyp alleviating myocardial IRI by promoting mitochondrial fusion via activating the Stat3-Tom70-Opa1 pathway.

Hyp alleviating myocardial IRI by promoting mitochondrial fusion via activating the Stat3-Tom70-Opa1 pathway (Figure 9). These findings suggest that Hyp or promotion of mitochondrial fusion may become a new therapeutic option for the prevention and treatment of myocardial IRI in heart transplantation.

## Data Availability

The original contributions presented in the study are included in the article/supplementary material, further inquiries can be directed to the corresponding authors.

## Glossary

IRI: ischemia–reperfusion injury
Hyp: hyperoside
Tom: translocase of mitochondrial outer membrane
Drp1: dynamin-related protein 1
Opa1: optic atrophy 1
AAV: adeno-associated virus
MDA: malondialdehyde
SOD: superoxide dismutase
GPX: glutathione peroxidase
BCA: bicinchoninic acid
IL-6: interleukin-6
MCP-1: monocyte chemoattractant protein-1
TNF-α: tumor necrosis factor-α
LDH: lactate dehydrogenase
cTnI: cardiac troponin I
ELISA: enzyme-linked immunosorbent assay
NADP: nicotinamide adenine dinucleotide phosphate
NADPH: nicotinamide adenosine dinucleotide phosphate
GSSG: glutathione oxidized form
GSH: L-Glutathione
CS: citrate synthase
IDH: isocitrate dehydrogenase
RIPA: radioimmunoprecipitation assay
p-Stat3: phosphorylated Stat3
Bax: Bcl-2 associated X protein
Bcl-2: B cell lymphoma/lewkmia-2
GAPDH: Glyceraldehyde-3-phosphate dehydrogenase
Mfn1: mitofusin 1
Mfn2: mitofusin 2
UW: University of Wisconsin
NRCMs: neonatal rat cardiomyocytes
DMEM: Dulbecco’s modified Eagle’s medium
FBS: fetal bovine serum
H/R: hypoxia–reoxygenation
MOI: multiplicity of infection
TUNEL: terminal deoxynucleotidyl transferase dUTP nick end labeling
siRNA: Short interfering RNA
DHE: dihydroethidium
Co-IP: Co-immunoprecipitation
TEM: transmission electron microscopy
MICU1: mitochondrial calcium uptake 1

## References

[B1] Araiso Y. Imai K. Endo T. (2022). Role of the tom complex in protein import into mitochondria: structural views. Annu. Rev. Biochem. 91, 679–703. 10.1146/annurev-biochem-032620-104527 35287471

[B2] Backes S. Hess S. Boos F. Woellhaf M. W. Gödel S. Jung M. (2018). Tom70 enhances mitochondrial preprotein import efficiency by binding to internal targeting sequences. J. Cell Biol. 217 (4), 1369–1382. 10.1083/jcb.201708044 29382700 PMC5881500

[B3] Backes S. Bykov Y. S. Flohr T. Räschle M. Zhou J. Lenhard S. (2021). The chaperone-binding activity of the mitochondrial surface receptor Tom70 protects the cytosol against mitoprotein-induced stress. Cell Rep. 35 (1), 108936. 10.1016/j.celrep.2021.108936 33826901 PMC7615001

[B4] Benke K. Jász D. K. Szilágyi Á. L. Baráth B. Tuboly E. Márton A. R. (2021). Methane supplementation improves graft function in experimental heart transplantation. J. Heart Lung Transpl. 40 (3), 183–192. 10.1016/j.healun.2020.11.003 33277170

[B5] Cai S. Ichimaru N. Zhao M. Fujino M. Ito H. Ota U. (2016). Prolonged mouse cardiac graft cold storage *via* attenuating ischemia-reperfusion injury using a new antioxidant-based preservation solution. Transplantation 100 (5), 1032–1040. 10.1097/TP.0000000000001079 26845308

[B6] Capuzzimati M. Hough O. Liu M. (2022). Cell death and ischemia-reperfusion injury in lung transplantation. J. Heart Lung Transpl. 41 (8), 1003–1013. 10.1016/j.healun.2022.05.013 35710485

[B7] Chambers D. C. Yusen R. D. Cherikh W. S. Goldfarb S. B. Kucheryavaya A. Y. Khusch K. (2017). The registry of the international society for heart and lung transplantation: thirty-fourth adult lung and heart-lung transplantation report-2017; focus theme: allograft ischemic time. J. Heart Lung Transpl. 36 (10), 1047–1059. 10.1016/j.healun.2017.07.016 28784324

[B8] Charachit N. Sukhamwang A. Dejkriengkraikul P. Yodkeeree S. (2022). Hyperoside and quercitrin in Houttuynia cordata extract attenuate UVB-induced human keratinocyte cell damage and oxidative stress *via* modulation of MAPKs and akt signaling pathway. Antioxidants 11 (2), 221. 10.3390/antiox11020221 35204104 PMC8868276

[B9] Chen Z. An X. Liu X. Qi J. Ding D. Zhao M. (2017). Hyperoside alleviates adriamycin-induced podocyte injury *via* inhibiting mitochondrial fission. Oncotarget 8 (51), 88792–88803. 10.18632/oncotarget.21287 29179476 PMC5687646

[B10] Chen L. Qin Z. Ruan Z. B. (2023). Hyperoside alleviates doxorubicin-induced myocardial cells apoptosis by inhibiting the apoptosis signal-regulating kinase 1/p38 pathway. PeerJ 11, e15315. 10.7717/peerj.15315 37220525 PMC10200097

[B11] Choi J. H. Kim D. W. Yun N. Choi J. S. Islam M. N. Kim Y. S. (2011). Protective effects of hyperoside against carbon tetrachloride-induced liver damage in mice. J. Nat. Prod. 74 (5), 1055–1060. 10.1021/np200001x 21428416

[B12] Coulson M. T. Jablonski P. Howden B. O. Thomson N. M. Stein A. N. (2005). Beyond operational tolerance: effect of ischemic injury on development of chronic damage in renal grafts. Transplantation 80 (3), 353–361. 10.1097/01.tp.0000168214.84417.7d 16082331

[B13] Ding M. Liu C. Shi R. Yu M. Zeng K. Kang J. (2020). Mitochondrial fusion promoter restores mitochondrial dynamics balance and ameliorates diabetic cardiomyopathy in an optic atrophy 1‐dependent way. Acta Physiol. 229 (1), e13428. 10.1111/apha.13428 31840416

[B14] Du J. Li H. Song J. Wang T. Dong Y. Zhan A. (2022). AMPK activation alleviates myocardial ischemia-reperfusion injury by regulating Drp1-mediated mitochondrial dynamics. Front. Pharmacol. 13, 862204. 10.3389/fphar.2022.862204 35860026 PMC9289369

[B15] Endo T. Yamano K. (2009). Multiple pathways for mitochondrial protein traffic. Biol. Chem. 390 (8), 723–730. 10.1515/BC.2009.087 19453276

[B16] Fan A. C. Y. Gava L. M. Ramos C. H. I. Young J. C. (2010). Human mitochondrial import receptor Tom70 functions as a monomer. Biochem. J. 429 (3), 553–563. 10.1042/BJ20091855 20504278 PMC5026490

[B17] Filadi R. Leal N. S. Schreiner B. Rossi A. Dentoni G. Pinho C. M. (2018). Tom70 sustains cell bioenergetics by promoting IP3R3-mediated er to mitochondria ca2+ transfer. Curr. Biol. 28 (3), 369–382. 10.1016/j.cub.2017.12.047 29395920

[B18] Franco A. Dang X. Zhang L. Molinoff P. B. Dorn G. W. 2nd (2022). Mitochondrial dysfunction and pharmacodynamics of mitofusin activation in murine Charcot-Marie-Tooth disease type 2A. Pharmacol. Exp. Ther. 383 (2), 137–148. 10.1124/jpet.122.001332 36507849 PMC9553116

[B19] Han J. Xuan J. L. Hu H. R. Chen Z. W. (2015). Protective effect against myocardial ischemia reperfusion injuries induced by hyperoside preconditioning and its relationship with PI3K/Akt signaling pathway in rats. Zhongguo Zhong Yao Za Zhi 40 (1), 118–123. 25993800

[B20] Hansson P. C. Alikhani N. Behbahani H. Wiehager B. Pavlov P. F. Alafuzoff I. (2008). The amyloid beta-peptide is imported into mitochondria *via* the TOM import machinery and localized to mitochondrial cristae. Proc. Natl. Acad. Sci. U. S. A. 105 (35), 13145–13150. 10.1073/pnas.0806192105 18757748 PMC2527349

[B21] Hou J. Liu Y. Liu L. Li X. M. (2016). Protective effect of hyperoside on cardiac ischemia reperfusion injury through inhibition of ER stress and activation of Nrf2 signaling. Asian Pac J. Trop. Med. 9 (1), 76–80. 10.1016/j.apjtm.2015.12.001 26851792

[B22] Hou J. Wang X. Li Y. Hou J. Li X. Zhang X. (2022). Positive regulation of endothelial Tom70 by metformin as a new mechanism against cardiac microvascular injury in diabetes. Mitochondrion 65, 150–160. 10.1016/j.mito.2022.06.005 35779798

[B23] Jiang S. Liu S. Hou Y. Lu C. Yang W. Ji T. (2022). Cardiac-specific overexpression of Claudin-5 exerts protection against myocardial ischemia and reperfusion injury. BBA-Mol Basis Dis. 1868 (12), 166535. 10.1016/j.bbadis.2022.166535 36058416

[B24] John M. M. Shih W. Estevez D. Martens T. P. Bailey L. L. Razzouk A. J. (2019). Interaction between ischemic time and donor age on adult heart transplant outcomes in the modern era. Ann. Thorac. Surg. 108 (3), 744–748. 10.1016/j.athoracsur.2019.03.042 30986413

[B25] Kreimendahl S. Rassow J. (2020). The mitochondrial outer membrane protein Tom70-mediator in protein traffic, membrane contact sites and innate immunity. Int. J. Mol. Sci. 21 (19), 7262. 10.3390/ijms21197262 33019591 PMC7583919

[B26] Ku S. K. Zhou W. Lee W. Han M. S. Na M. Bae J. S. (2015). Anti-inflammatory effects of hyperoside in human endothelial cells and in mice. Inflammation 38 (2), 784–799. 10.1007/s10753-014-9989-8 25097077

[B27] Kwon J. H. Huckaby L. V. Sloan B. Pope N. H. Witer L. J. Tedford R. J. (2022). Prolonged ischemia times for heart transplantation: impact of the 2018 allocation change. Ann. Thorac. Surg. 114 (4), 1386–1394. 10.1016/j.athoracsur.2022.02.029 35247342

[B28] Latorre-Muro P. O Malley K. E. Bennett C. F. Perry E. A. Balsa E. Tavares C. D. J. (2021). A cold-stress-inducible PERK/OGT axis controls TOM70-assisted mitochondrial protein import and cristae formation. Cell Metab. 33 (3), 598–614.e7. 10.1016/j.cmet.2021.01.013 33592173 PMC7962155

[B29] Li W. Liu M. Xu Y. F. Feng Y. Che J. P. Wang G. C. (2014a). Combination of quercetin and hyperoside has anticancer effects on renal cancer cells through inhibition of oncogenic microRNA-27a. Oncol. Rep. 31 (1), 117–124. 10.3892/or.2013.2811 24173369

[B30] Li J. Qi M. Li C. Shi D. Zhang D. Xie D. (2014b). Tom70 serves as a molecular switch to determine pathological cardiac hypertrophy. Cell Res. 24 (8), 977–993. 10.1038/cr.2014.94 25022898 PMC4123302

[B31] Liu X. Wei B. Shi H. Shan Y. F. Wang C. (2010). Tom70 mediates activation of interferon regulatory factor 3 on mitochondria. Cell Res. 20 (9), 994–1011. 10.1038/cr.2010.103 20628368

[B32] Liu R. Xiong Q. Shu Q. Wu W. N. Cheng J. Fu H. (2012). Hyperoside protects cortical neurons from oxygen–glucose deprivation–reperfusion induced injury *via* nitric oxide signal pathway. Brain Res. 1469, 164–173. 10.1016/j.brainres.2012.06.044 22771858

[B33] Liu Q. Chang C. E. Wooldredge A. C. Fong B. Kennedy B. K. Zhou C. (2022). Tom70-based transcriptional regulation of mitochondrial biogenesis and aging. Elife 11, e75658. 10.7554/eLife.75658 35234609 PMC8926401

[B34] Loganathan S. Korkmaz-Icoz S. Radovits T. Li S. Mikles B. Barnucz E. (2015). Effects of soluble guanylate cyclase activation on heart transplantation in a rat model. J. Heart Lung Transpl. 34 (10), 1346–1353. 10.1016/j.healun.2015.05.006 26210750

[B35] Maneechote C. Palee S. Kerdphoo S. Jaiwongkam T. Chattipakorn S. C. Chattipakorn N. (2019). Balancing mitochondrial dynamics *via* increasing mitochondrial fusion attenuates infarct size and left ventricular dysfunction in rats with cardiac ischemia/reperfusion injury. Clin. Sci. 133 (3), 497–513. 10.1042/CS20190014 30705107

[B36] Maneechote C. Palee S. Kerdphoo S. Jaiwongkam T. Chattipakorn S. C. Chattipakorn N. (2022). Modulating mitochondrial dynamics attenuates cardiac ischemia-reperfusion injury in prediabetic rats. Acta Pharmacol. Sin. 43 (1), 26–38. 10.1038/s41401-021-00626-3 33712720 PMC8724282

[B37] Moskowitzova K. Shin B. Liu K. Ramirez-Barbieri G. Guariento A. Blitzer D. (2019). Mitochondrial transplantation prolongs cold ischemia time in murine heart transplantation. J. Heart Lung Transpl. 38 (1), 92–99. 10.1016/j.healun.2018.09.025 30391192 PMC6574228

[B38] Neupert W. Herrmann J. M. (2007). Translocation of proteins into mitochondria. Annu. Rev. Biochem. 76, 723–749. 10.1146/annurev.biochem.76.052705.163409 17263664

[B39] Pei H. F. Hou J. N. Wei F. P. Xue Q. Zhang F. Peng C. F. (2017). Melatonin attenuates postmyocardial infarction injury *via* increasing Tom70 expression. J. Pineal Res. 62 (1), e12371. 10.1111/jpi.12371 27706848

[B40] Pfanner N. Warscheid B. Wiedemann N. (2019). Mitochondrial proteins: from biogenesis to functional networks. Nat. Rev. Mol. Cell Biol. 20 (5), 267–284. 10.1038/s41580-018-0092-0 30626975 PMC6684368

[B41] Shi Y. Qiu X. Dai M. Zhang X. Jin G. (2019). Hyperoside attenuates hepatic ischemia-reperfusion injury by suppressing oxidative stress and inhibiting apoptosis in rats. Transpl 51 (6), 2051–2059. 10.1016/j.transproceed.2019.04.066 31399183

[B42] Smith S. F. Hosgood S. A. Nicholson M. L. (2019). Ischemia-reperfusion injury in renal transplantation: 3 key signaling pathways in tubular epithelial cells. Kidney Int. 95 (1), 50–56. 10.1016/j.kint.2018.10.009 30606429

[B43] Tang P. C. Lei I. Chen Y. E. Wang Z. Ailawadi G. Romano M. A. (2021). Risk factors for heart transplant survival with greater than 5 h of donor heart ischemic time. J. Card. Surg. 36 (8), 2677–2684. 10.1111/jocs.15621 34018246 PMC11175709

[B44] Valero-Masa M. J. Gonzalez-Vilchez F. Almenar-Bonet L. Crespo-Leiro M. G. Manito-Lorite N. Sobrino-Márquez J. M. (2020). Cold ischemia >4 hours increases heart transplantation mortality. An analysis of the Spanish heart transplantation registry. Int. J. Cardiol. 319, 14–19. 10.1016/j.ijcard.2020.06.009 32569699

[B45] Vongsfak J. Pratchayasakul W. Apaijai N. Vaniyapong T. Chattipakorn N. Chattipakorn S. C. (2021). The alterations in mitochondrial dynamics following cerebral ischemia/reperfusion injury. Antioxidants 10 (9), 1384. 10.3390/antiox10091384 34573016 PMC8468543

[B46] Wang Q. Wei H. C. Zhou S. J. Li Y. Zheng T. T. Zhou C. Z. (2022). Hyperoside: a review on its sources, biological activities, and molecular mechanisms. Phytother. Res. 36 (7), 2779–2802. 10.1002/ptr.7478 35561084

[B47] Wang X. O'Brien M. E. Yu J. Xu C. Zhang Q. Lu S. (2019). Prolonged cold ischemia induces necroptotic cell death in ischemia-reperfusion injury and contributes to primary graft dysfunction after lung transplantation. Am. J. Respir. Cell Mol. Biol. 61 (2), 244–256. 10.1165/rcmb.2018-0207OC 30742487 PMC6670033

[B48] Wang P. Wang D. Yang Y. Hou J. Wan J. Ran F. (2020). Tom70 protects against diabetic cardiomyopathy through its antioxidant and antiapoptotic properties. Hypertens. Res. 43 (10), 1047–1056. 10.1038/s41440-020-0518-x 32724135

[B49] Wang Y. Wang Z. Dong C. Yim W. Y. Liu Z. Hou J. (2025). Initial cardioplegic flush with crystalloid cardioplegia improves donor heart preservation and function *via* NR4A3 upregulation and metabolic reprogramming. Sci. Bull. 70, 1673–1690. 10.1016/j.scib.2025.03.019 40190001

[B50] Wu L. Li Q. Liu S. An X. Huang Z. Zhang B. (2019). Protective effect of hyperoside against renal ischemia-reperfusion injury *via* modulating mitochondrial fission, oxidative stress, and apoptosis. Free Radic. Res. 53 (7), 727–736. 10.1080/10715762.2019.1623883 31130024

[B51] Xue Q. Pei H. Liu Q. Zhao M. Sun J. Gao E. (2017). MICU1 protects against myocardial ischemia/reperfusion injury and its control by the importer receptor Tom70. Cell Death Dis. 8 (7), e2923. 10.1038/cddis.2017.280 28703803 PMC5550843

[B52] Zhai Y. Petrowsky H. Hong J. C. Busuttil R. W. Kupiec-Weglinski J. W. (2013). Ischaemia-reperfusion injury in liver transplantation-from bench to bedside. Nat. Rev. Gastro Hepat. 10 (2), 79–89. 10.1038/nrgastro.2012.225 23229329 PMC3577927

[B54] Zheng H. Su Y. Zhu C. Quan D. Skaro A. I. McAlister V. (2021). An addition of U0126 protecting heart grafts from prolonged cold ischemia-reperfusion injury in heart transplantation: a new preservation strategy. Transplantation 105 (2), 308–317. 10.1097/TP.0000000000003402 32776778

[B55] Zhu C. Su Y. Juriasingani S. Zheng H. Veramkovich V. Jiang J. (2019). Supplementing preservation solution with mitochondria-targeted H2 S donor AP39 protects cardiac grafts from prolonged cold ischemia-reperfusion injury in heart transplantation. Am. J. Transpl. 19 (11), 3139–3148. 10.1111/ajt.15539 31338943

[B56] Zhuang S. Xia S. Huang P. Wu J. Qu J. Chen R. (2021). Targeting P2RX1 alleviates renal ischemia/reperfusion injury by preserving mitochondrial dynamics. Pharmacol. Res. 170, 105712. 10.1016/j.phrs.2021.105712 34091010

